# Low PTGDS Expression Facilitates HNSCC by Suppressing Programmed Cell Death and Reducing B Cell–Mediated Immune Responses

**DOI:** 10.1155/mi/4521847

**Published:** 2026-03-22

**Authors:** Dan Tao, Yuan Zhong, Haoran Zhu, Zhenxing Zhang

**Affiliations:** ^1^ Department of Stomatology, Taizhou Central Hospital (Taizhou University Hospital), Taizhou, 318000, Zhejiang, China, tzc.edu.cn; ^2^ Xi’an Jiaotong University Health Science Center, Xi’an, 710000, Shaanxi, China, xjtu.edu.cn; ^3^ Department of Maxillofacial Surgery, Taizhou Central Hospital (Taizhou University Hospital), Taizhou, 318000, Zhejiang, China, tzc.edu.cn

**Keywords:** B cells, CAFs, HNSCC, immunotherapy, PCD

## Abstract

Head and neck squamous cell carcinoma (HNSCC) constitutes a significant global disease burden. Evasion of programmed cell death (PCD) is a hallmark of HNSCC progression. Cancer‐associated fibroblasts (CAFs) are a major cellular component of the HNSCC tumor microenvironment (TME). However, the mechanism by which CAFs contribute to PCD resistance remains poorly understood. This study included 24 single‐cell sequencing samples, the Cancer Genome Atlas (TCGA) data, and spatial transcriptome data from HNSCC samples. Single‐cell data were clustered using the Seurat package, and pseudotemporal trajectory analysis of CAF subsets was performed with Monocle2. The random forest algorithm was used to assess the prognostic importance of candidate genes. The ssGSEA and CIBERSORT algorithms were used to assess immune cell infiltration patterns in the TCGA samples. Gain‐of‐function assays, Western blotting, and immunohistochemistry were conducted to validate the biological functions of key targets. Here, we identified seven CAF subsets and clarified their potential differentiation directions. Thirty‐two CAF‐associated PCD regulators with prognostic significance were identified, among which, prostaglandin D2 synthase (PTGDS) and squalene epoxidase (SQLE) are the most important genes. PTGDS is significantly downregulated in HNSCC and associated with favorable prognosis. PTGDS overexpression significantly inhibited clonogenicity, proliferation, and invasion while promoting apoptosis in HNSCC cells (*p* < 0.05). In addition, PTGDS expression is significantly enriched in B cell–related pathways. Mechanistically, PTGDS overexpression increased chemokine (C‐X‐C motif) ligand 13 (CXCL13) expression and enhanced immune cell infiltration. These findings identified PTGDS as a central regulator linking CAF‐mediated PCD resistance and B cell immune modulation in HNSCC, suggesting its potential as both a diagnostic biomarker and therapeutic target.

## 1. Introduction

Head and neck squamous cell carcinoma (HNSCC) is the most common pathological type of head and neck cancer and the sixth most prevalent cancer worldwide, with over 750,000 new cases diagnosed annually, representing a major medical and socioeconomic challenge [1]. Current mainstream treatments for HNSCC include radical resection, radiotherapy, chemotherapy, and targeted therapy. Despite advances in treatment and drug development, the absence of reliable molecular biomarkers, combined with the rapid growth, aggressiveness, and high lymph node metastasis rate of HNSCC, results in most patients being diagnosed at advanced stages, leading to poor long‐term survival and quality of life, with a 5‐year survival rate of ~50% [2]. Therefore, identifying accurate early diagnostic biomarkers and clarifying the molecular mechanisms underlying HNSCC progression are essential for improving early detection, patient outcomes, and reducing the overall disease burden.

Some patients with rapidly progressing tumors and poor long‐term prognosis exhibit significant resistance to chemotherapy, radiotherapy, and immunotherapy. This is largely attributed to the heterogeneity of solid tumors and the complex regulatory mechanisms of the tumor microenvironment (TME). The TME is a complex and dynamic system composed of cellular and noncellular components—including tumor cells, stromal cells, and immune cells—that plays a pivotal role in tumor development, progression, and treatment response [3–5]. Cancer‐associated fibroblasts (CAFs) are a core component of the solid tumor TME. CAFs are typically the most abundant type of stromal cell, and their presence is closely associated with poor prognosis, treatment resistance, and disease recurrence in various cancers [6]. CAFs promote HNSCC development through diverse mechanisms such as extracellular matrix remodeling, angiogenesis, radiotherapy resistance, inhibition of immune infiltration, and facilitation of immune evasion [7]. Increasing evidence suggests that depleting CAFs, reprogramming them into quiescent stromal cells, or targeting key proteins mediating interactions between CAFs, tumor cells, and immune cells may offer effective therapeutic strategies for HNSCC [8].

Programmed cell death (PCD) is a precisely regulated process in organisms, an active process regulated by genes, playing a central role in normal development, maintaining tissue homeostasis, resisting external stimuli, and inhibiting tumor formation [9]. A major characteristic of tumors is the suppression and dysregulation of PCD [10]. Recent studies have confirmed that CAFs can exert their oncogenic effects by inhibiting PCD. For example, activated CAFs can confer a survival advantage to tumor cells by inhibiting interferon‐induced p53‐dependent apoptosis [11]; interleukin‐6 (IL‐6) secreted by CAFs leads to apoptosis resistance in colorectal cancer cells [12]. Various therapeutic approaches, including radiotherapy and chemotherapy, are closely related to the induction of PCD in tumor cells [13].

Prostaglandin D2 synthase (PTGDS), also known as lipid transporter prostaglandin D synthase (L‐PGDS), is an essential enzyme involved in the cyclooxygenase (COX) pathway that generates bioactive metabolites from arachidonic acid [14]. As a functional protein, PTGDS catalyzes the production of prostaglandin D2 (PGD2), transports lipophilic substances, and participates in lysosome‐dependent PCD. PTGDS plays a crucial role in vascular regulation; its deficiency is closely associated with increased vascular permeability, angiogenesis, and endothelial‐mesenchymal transition [15, 16]. Although PTGDS downregulation has been associated with tumor progression in lung and gastric cancers [17, 18], its role in HNSCC pathogenesis has not yet been elucidated.

Using bioinformatics, machine learning, in vitro experiments, and HNSCC patient samples, this study confirmed that PTGDS expression decreases during CAF differentiation and may serve as a regulatory target by which CAFs inhibit PCD in tumor cells. This study preliminarily revealed the potential mechanism of the PTGDS‐CXCL13 axis in regulating B lymphocyte immune infiltration in HNSCC and validated the role of PTGDS in early diagnosis and prognosis of HNSCC.

## 2. Materials and Methods

### 2.1. Data Source

This study included 24 single‐cell sequencing samples of HNSCC from three studies (GSE139324, GSE173647, and GSE173964). Bulk RNA‐sequencing data of HNSCC were obtained from UCSC Xena (https://xena.ucsc.edu/). Spatial transcriptome data were obtained from GSE181300.

### 2.2. Quality Control, Cell Type Clustering, and Identification of Major Cell Types

Cells with mitochondrial gene content exceeding 20% were excluded, leaving 73,660 cells for downstream analysis. Because batch effects were minimal across samples, they were not corrected to preserve genuine biological variation among cell populations. We used Seurat to first standardize and normalize the expression matrix, then applied the FindVariable function to select the top 2000 variable genes, and performed principal component analysis [19]. After clustering the data, we annotated the cells according to cell characteristics provided by the CellTypist database (https://www.celltypist.org/).

### 2.3. Cell Subpopulation Identification

We extracted the expression matrix of the fibroblast population for further analysis, using the FindVariable function to select the top 2000 variable genes and performing normalization and principal component analysis. After using the FindAllMarkers function to identify differentially expressed genes among different cell populations, consistent with our previous research, we ultimately identified seven tumor‐associated fibroblast subsets [20].

### 2.4. Pseudotemporal Trajectory Analysis

One hundred thirteen genes (*p* < 0.01) from 1255 PCD‐related genes had an impact on the prognosis of HNSCC patients [21]. Monocle2 package was used to perform pseudotemporal trajectory analysis on the CAF subsets [22]. Monocle2‐selected hypervariable genes were subjected to dimensionality reduction, followed by differentialGeneTest analysis to determine the influence of the 113 PCD‐related genes on CAF differentiation (*q* < 1 × 10^−6^). Finally, 32 genes that affected both CAF differentiation and the prognosis of HNSCC patients were selected for further analysis.

### 2.5. Random Forest Algorithm for Assessing the Importance of Genes to Patient Prognosis

Expression matrices of the 32 candidate genes, together with patient survival data, were used to construct a random forest model that ranked gene importance for HNSCC prognosis [23]. Survival curves were plotted using the survival test. A nonparametric rank‐sum test was used to evaluate the significance of gene expression differences between normal and cancerous tissues.

### 2.6. Spatial Transcriptome Observation of Gene Expression and Spatial Distribution

STUtility was used for quality control and integration of spatial transcriptome samples, and the FeatureOverlay function was used to observe the expression and spatial distribution of target genes on HNSCC slices [24].

### 2.7. TME Analysis of the Cancer Genome Atlas (TCGA) Samples

The ssGSEA algorithm implemented in the GSVA R package was used to predict the types and relative proportions of infiltrating immune cells in TCGA samples. The immune scoring gene set used by ssGSEA is derived from “Local mutational diversity drives intratumoral immune heterogeneity in non‐small cell lung cancer” [25, 26].

### 2.8. Functional Exploration of PTGDS and Squalene Epoxidase (SQLE)

The transcripts per million (TPM) expression data of PTGDS and SQLE were processed using log2(*x* + 1), and the Pearson correlation coefficient was calculated. NMF was used to cluster 501 samples containing only PTGDS and SQLE gene expression levels, dividing the samples into two classes. t‐Distributed stochastic neighbor embedding (t‐SNE) dimensionality reduction was then performed using the tinyarray R package. Type C1 was characterized by low SQLE expression and high PTGDS expression, while type C2 was characterized by high SQLE expression and low PTGDS expression. Differential gene analysis between C1 and C2 was performed using the limma package, with |log2FC| > 1 and adjusted *p* < 0.05 as the differential gene filtering criteria. Kyoto Encyclopedia of Genes and Genomes (KEGG) was used for differential gene enrichment analysis between type C1 and C2 patients. CIBERSORT was used to perform deconvolution on the samples to estimate the relative abundance of different immune cell populations, and bar charts were generated for visualization.

### 2.9. Cell Line Culture and Treatment

This study used the HNSCC cell lines Centre Antoine Lacassagne‐27 (Cal 27) and Human Tongue Squamous Cell Carcinoma Cell Line‐9 (SCC 9), purchased from the American Type Culture Collection (ATCC), and identified them using the short tandem repeat (STR) method. HNSCC cells were cultured in Dulbecco’s modified Eagle’s medium/F12 (DMEM/F12) containing 10% fetal bovine serum (FBS), 100 U/mL penicillin, and 100 μg/mL streptomycin and stored in a humidified incubator at 37°C with 5% CO_2_. Plasmids were synthesized by GeneChem (Shanghai GeneChem Co., Ltd.). Following official instructions, the PTGDS overexpression plasmid (K25K0699) and its negative control (CON468) were transfected using E‐Trans. After the transfected cells emitted green fluorescence under a fluorescence microscope, the PTGDS expression level in tumor cells was detected using quantitative reverse transcription polymerase chain reaction (qRT‐PCR). Total RNA was extracted using TRIzol reagent. Glyceraldehyde 3‐phosphate dehydrogenase was used as an internal reference. Two primers were purchased from GeneCopoeia.

### 2.10. Cell Counting Kit 8 (CCK8) Experiment

PTGDS‐overexpressing and control cells were seeded into 96‐well plates at a density of 2 × 10^3^ cells per well. At 24, 48, and 72 h, 10 μL of CCK‐8 reagent was added to each well, and incubation continued for 2 h. The OD value of all samples at 450 nm was measured using a microplate reader.

### 2.11. Plate Cloning Experiment

HNSCC cells from each group were collected and trypsinized and then subjected to 1500 rpm centrifugation. For monolayer cultures, cells (1 × 10^3^/well) were seeded in a 3 cm plate for 14 days. Colony fixation and staining were performed before imaging and enumeration.

### 2.12. Scratch and Intrusion Tests

A scratch assay was performed to evaluate the impact of PTGDS on HNSCC cell migration. Cells were seeded in 6‐well plates and cultured until ~80% confluence. They were then transfected with the overexpression plasmid and a control plasmid. After 24 h of further culture, scratches were artificially created on the confluent cell monolayer using a sterile pipette tip (20 µL). After washing with PBS, images were taken. The FBS concentration in the culture medium was further reduced to 1%, and incubation continued. Wound images were taken again after 24 h to assess the tumor cell migration ability.

Tumor cell invasion was assessed using a Transwell invasion assay (24 wells). A total of 2 × 10^5^ cells were suspended in serum‐free medium and seeded onto the upper layer of Matrigel‐coated Transwell chambers. Complete culture medium was placed in the lower layer of the chambers to stimulate tumor cell invasion. After 12 h of incubation, cells that had invaded below the membrane surface were washed and stained with crystal violet solution and counted.

### 2.13. Apoptosis Identification

Tumor cells were collected and digested with trypsin. Once the cells became rounded, they were immediately added to the culture medium and centrifuged. After washing twice with precooled PBS, the Annexin VFITC/PI Apoptosis Detection Kit (KeyGEN) was used to detect apoptotic cells according to the manufacturer’s instructions. Fluorescence signals were recorded using flow cytometry (BD Biosciences).

### 2.14. Western Blotting

Harvested HNSCC cells were lysed on ice for 30 min using RIPA buffer supplemented with 100 mM benzyl sulfonyl fluoride. The supernatant was subjected to sodium dodecyl sulfate‐polyacrylamide gel electrophoresis, and the separated proteins were transferred to polyvinylidene fluoride membranes. Membrane blocking was performed using 5% nonfat milk, followed by overnight incubation at 4°C with primary antibodies PTGDS (Proteintech), CXCL13 (XY‐Bioscience), and actin (CST). The membranes incubated with the secondary peroxidase‐conjugated antibodies were visualized using chemiluminescence reagents (LAS 4000 mini) according to the manufacturer’s instructions.

### 2.15. Patient Cohort and Immunohistochemistry

For immunohistochemical staining and follow‐up, paraffin‐embedded surgical tissue specimens from 50 HNSCC patients used to detect PTGDS and CXCL13 expression in this study were obtained from our hospital between 2017 and 2019. All patients in this study met the following criteria: They underwent surgical resection at our hospital, and pathological examination confirmed the specimens as primary HNSCC; they had not received preoperative radiotherapy or chemotherapy; they signed informed consent forms; they had follow‐up records. All procedures involving human participants in this study complied with the 1964 Declaration of Helsinki and its subsequent amendments or similar ethical standards. This study was also approved by our institution’s research committee ethical standards (2020KT01). Primary tumor size and stage were determined according to the tumor‐node‐metastases (TNM) classification system of the International Union for Cancer Control. Tumor histological grading was performed according to the Broder classification system. Paraffin‐embedded specimens were cut into 4‐μm‐thick sections. Antigen retrieval was performed by heating with citrate buffer. Endogenous peroxidase was blocked by incubation with 3% H_2_O_2_. Sections were incubated overnight with primary antibodies against PTGDS and CXCL13. Binding antibodies were detected using a Polink2‐plus kit. The reaction products were visualized using 3,3‐diaminobenzidine solution. Experienced pathologists performed a blinded scoring system based on staining intensity and degree (H‐score). Patients were categorized as high‐ or low‐expression based on the median score.

### 2.16. Statistical Analysis

All data analyses were performed using R software. Proliferation, invasion, migration, apoptosis of different cell types, and relative expression levels of all proteins were analyzed using independent samples *t*‐tests. Survival analysis was performed using the Log‐rank test. Correlation analysis was performed using the chi‐square test. *p* < 0.05 was defined as statistically significant.

## 3. Results

### 3.1. 32 CAF‐Related PCD Regulatory Genes Were Identified

Twenty‐four single‐cell sequencing samples of HNSCC were collected. After standardizing the expression matrices, we performed dimensionality reduction annotation on the obtained data. Six cell populations were identified (T/NK cells, myeloid cells, epithelial cells, fibroblasts, B cells, and mast cells). Among them, the epithelial cell population, fibroblast population, and B cell population showed greater heterogeneity (Figure 1A, Supporting Information 1: Figure S1A). After extracting the fibroblast expression matrix, dimensionality reduction analysis identified seven fibroblast subpopulations, consistent with our previous findings (PENK + myCAFs, CKS2‐ iCAFs, PTN + myCAFs, CKS2+ iCAFs, CENPF‐/MYLPF‐ myCAFs, CENPF + myCAFs, and MYLPF + myCAFs) (Figure 1B, Supporting Information 1: Figure S1B) [20]. Pseudotemporal trajectory analysis revealed a differentiation trajectory among CAF subsets, identifying CENPF + myCAFs and CENPF‐/MYLPF‐ myCAFs as early progenitor populations, while CKS2 + iCAFs and PENK + myCAFs represented terminally differentiated subsets (Figure 1C,D). To more effectively screen for genes that significantly influence PCD in HNSCC, we first used single‐gene Cox regression to screen 113 genes with significant prognostic effects on HNSCC from 1255 PCD‐related genes. Subsequently, the differentialGeneTest function was used to assess the changes in these 113 genes during CAF differentiation. Thirty‐two genes with prognostic value and significantly different expression levels with CAF differentiation were designated as CAF‐related PCD regulatory genes and identified for subsequent analysis. Figure 1E shows the expression changes of these 32 genes during CAF differentiation. These genes initially exhibited three types of changes and were classified into three clusters. Cluster 1 has fewer genes, exhibiting low expression at the onset of differentiation, gradually increasing expression, and finally decreasing expression. Cluster 2 has the most genes, with overall expression gradually decreasing as CAF differentiate. Cluster 3, on the other hand, shows a significant increase in gene expression as CAF differentiate.

Figure 1Identification of 32 CAF‐related PCD regulatory genes in HNSCC. (A) Dimension reduction plot showing six major HNSCC cell populations. (B) Dimension reduction plot of the CAF population revealing seven fibroblast subtypes. (C–D) Pseudotemporal trajectory analysis, with darker colors indicating early and lighter colors indicating late differentiation stages. (E) Heatmap showing expression dynamics of 32 CAF‐related PCD regulatory genes along the differentiation trajectory. (F) Random forest analysis ranking the prognostic importance of these 32 genes. Abbreviations: CENPF, centromere protein F; CKS2, CDC28 protein kinase regulatory subunit 2; iCAFs, inflammatory CAFs; myCAFs, myofibroblasts; MYLPF, myosin light chain, phosphorylatable, fast skeletal muscle; PENK, proenkephalin; PTN, pleiotrophin.

**Figure:**
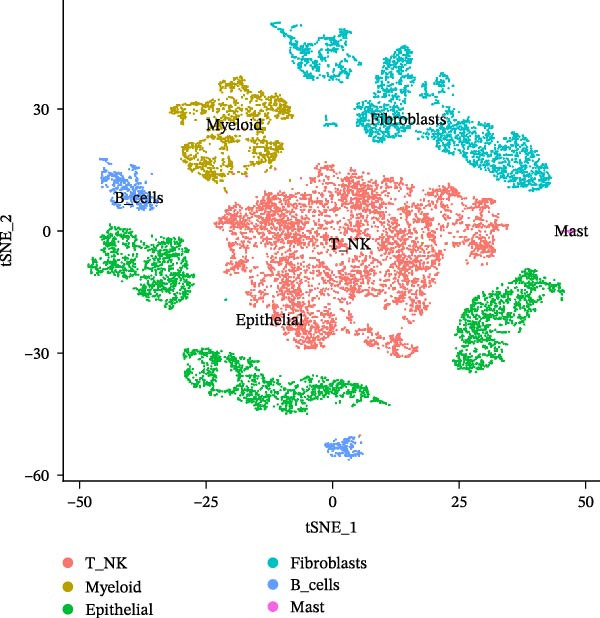
(A)

**Figure:**
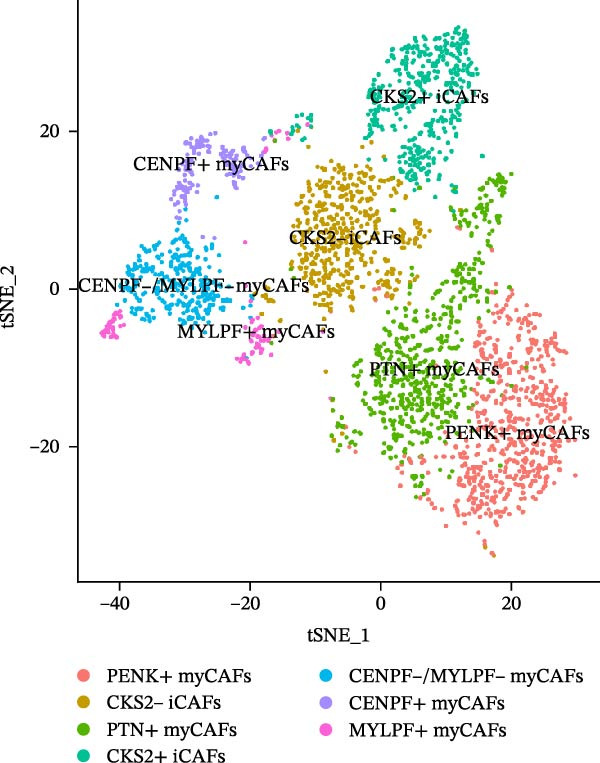
(B)

**Figure:**
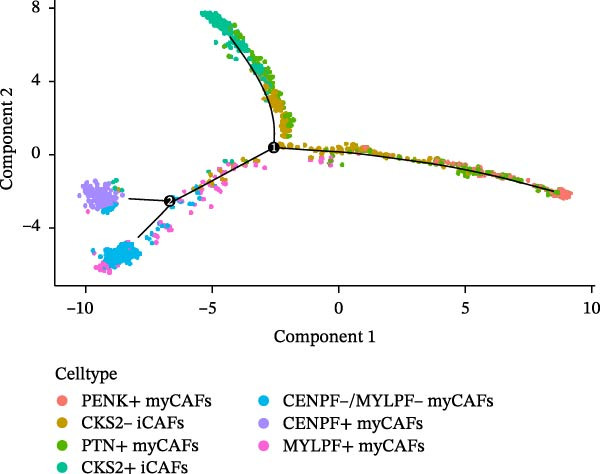
(C)

**Figure:**
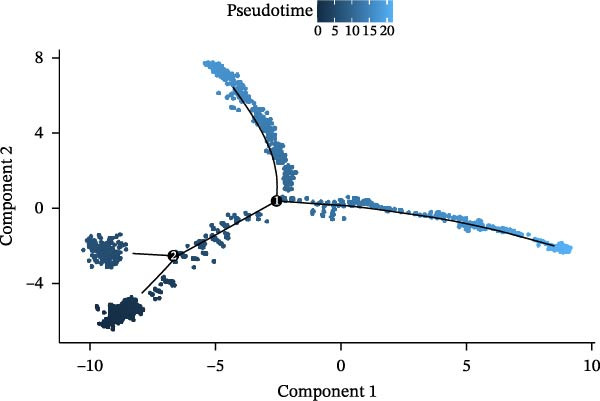
(D)

**Figure:**
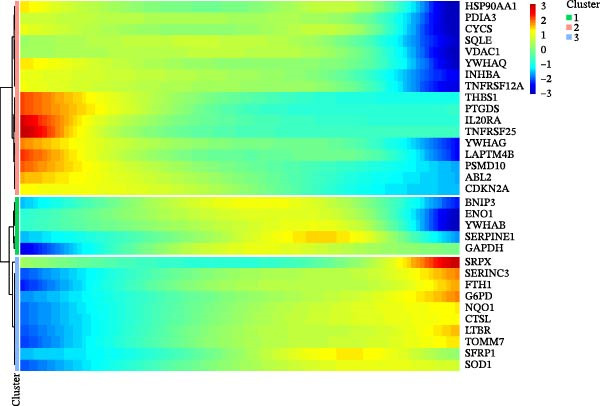
(E)

**Figure:**
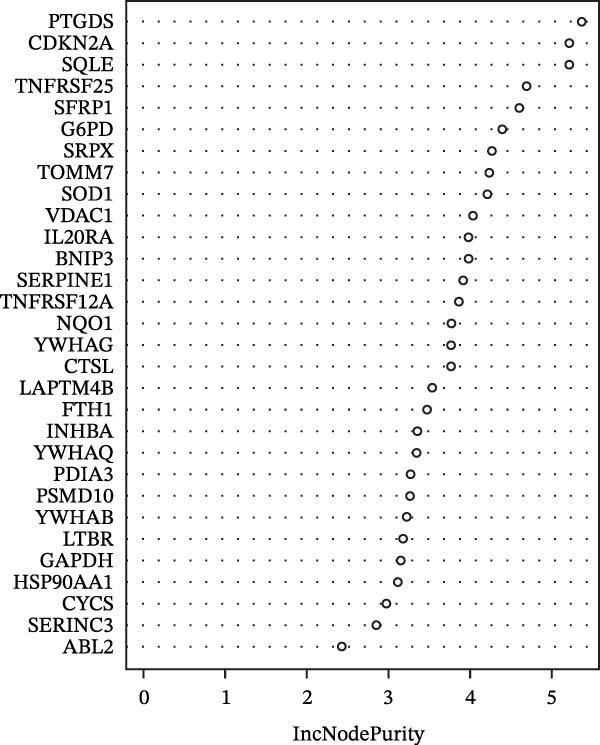
(F)

### 3.2. PTGDS and SQLE Were Closely Related to the Prognosis of HNSCC

A random forest model was constructed to calculate the gene importance for HNSCC prognosis. PTGDS, cyclin‐dependent kinase inhibitor 2A (CDKN2A), and SQLE were the three most important genes determining HNSCC prognosis (Figure 1F). We further incorporated TCGA data to analyze the expression and role of these three genes in HNSCC. In the TCGA dataset, PTGDS expression was markedly reduced in HNSCC tissues, whereas CDKN2A and SQLE were upregulated, suggesting potential opposing roles in tumor progression (Figure 2A). Furthermore, PTGDS and SQLE were closely associated with HNSCC prognosis. Patients with high PTGDS expression had a better prognosis. Conversely, patients with high SQLE expression had a worse prognosis. Furthermore, the results of multivariate Cox regression showed that PTGDS and SQLE had a predictive role (Table 1). However, CDKN2A expression did not show a significant difference in HNSCC prognosis (Figure 2B). Considering the central role of immune cells in the prognosis of HNSCC within the TME, we further divided patients into high and low expression groups based on the median expression of the three genes to assess the correlation between these genes and various cell populations in the immune microenvironment. The high PTGDS group was closely associated with greater infiltration of immune cells, including activated B cells, activated CD4 + T cells, activated CD8 + T cells, and NK cells. Conversely, high SQLE expression was associated with an immunosuppressive TME, marked by decreased infiltration of activated immune cells, including activated CD8 + T cells (Supporting Information 2: Figure S2A–C).

Figure 2Key CAF‐related PCD regulators and their clinical significance in HNSCC. (A) Expression levels of PTGDS, CDKN2A, and SQLE in TCGA HNSCC and normal tissues. (B) Kaplan–Meier survival curves showing the prognostic impact of the three genes. (C) Spatial transcriptomic mapping illustrating mutually exclusive expression patterns of PTGDS and SQLE in HNSCC sections.

**Figure:**
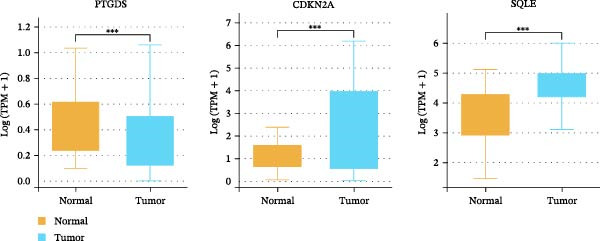
(A)

**Figure:**
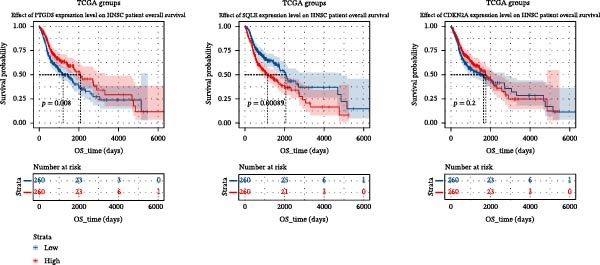
(B)

**Figure:**
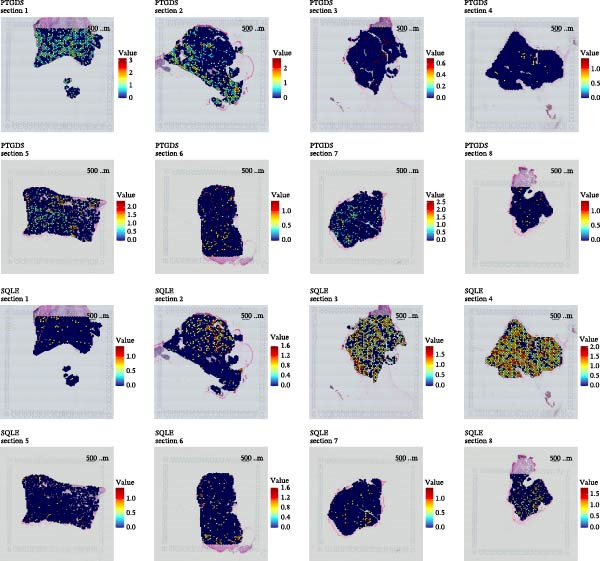
(C)

**Table 1:** Multivariate Cox regression analysis of key genes associated with overall survival in HNSCC patients.

Gene	Coefficient	HR	95% CI	*p*
PTGDS	−0.29	0.75	0.57–0.99	0.04
SQLE	0.45	1.56	1.19–2.06	<0.01

Spatial transcriptomic data were analyzed to examine the expression patterns and spatial distribution of PTGDS and SQLE in HNSCC tissue sections. PTGDS and SQLE showed significant mutual exclusivity in expression on the sections, especially in Sections 3, 4, and 5. High expression of PTGDS implied lower expression of SQLE (Figure 2C).

### 3.3. PTGDS and SQLE Were Closely Related to the Immune Microenvironment of HNSCC

We performed correlation analysis on the TPM expression data of PTGDS and SQLE. The results showed a significant negative correlation between PTGDS and SQLE (Figure 3A). Based on the expression levels of PTGDS and SQLE, we effectively divided TCGA patients into two groups: Group 1 (high expression of PTGDS and low expression of SQLE) and Group 2 (low expression of PTGDS and high expression of SQLE) (Figure 3B,C). Differentially expressed gene analysis was used to further clarify the potential roles of the two genes in HNSCC. We used |log2FC| > 1 and adjusted *p* < 0.05 as the differential gene filtering criteria and visualized the results using a volcano plot (Figure 3D). KEGG was used for enrichment analysis of differentially expressed genes. The results showed that differentially expressed genes in Group 1 patients were mainly enriched in immune cell‐related pathways, while those in Group 2 patients were mainly enriched in bacterial infection, estrogen signaling pathways, and mitogen‐activated protein kinase (MAPK) signaling pathways (Figure 3E). Group 1 highly expressed CD79A and CD79B, two B cell marker genes, as well as various immune‐related protein genes such as immunoglobulin heavy constant alpha 1 (IGHA1) and IGHG3. Furthermore, we observed significantly high expression of CXCL13 in population C1. We further used CIBERSORT to extrapolate the relative enrichment of different immune cell populations. Population C1 had a high abundance of immune cells, including B naive cells, plasma cells, and CD8 + T cells (Figure 3F).

Figure 3Functional characterization of PTGDS and SQLE in HNSCC. (A) Correlation between PTGDS and SQLE expression levels. (B–C) NMF clustering dividing TCGA HNSCC patients into two groups based on PTGDS and SQLE expression patterns. (D) Volcano plot of differentially expressed genes between the two groups. (E) KEGG pathway enrichment results. (F) CIBERSORT estimation of immune cell composition in the two groups.

**Figure:**
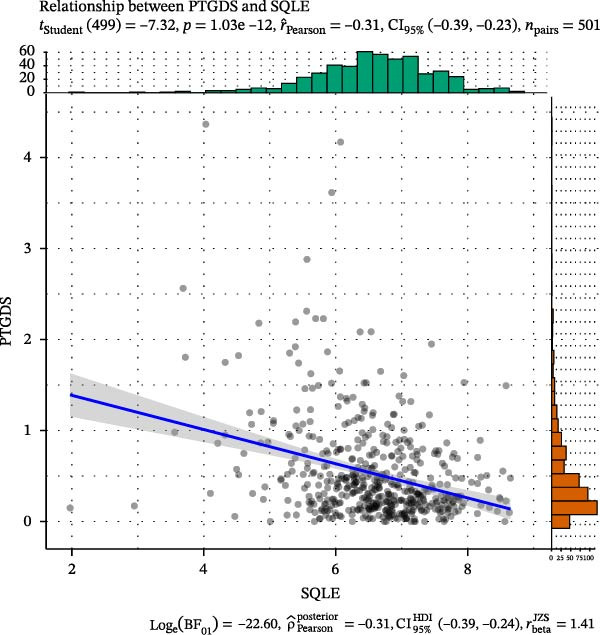
(A)

**Figure:**
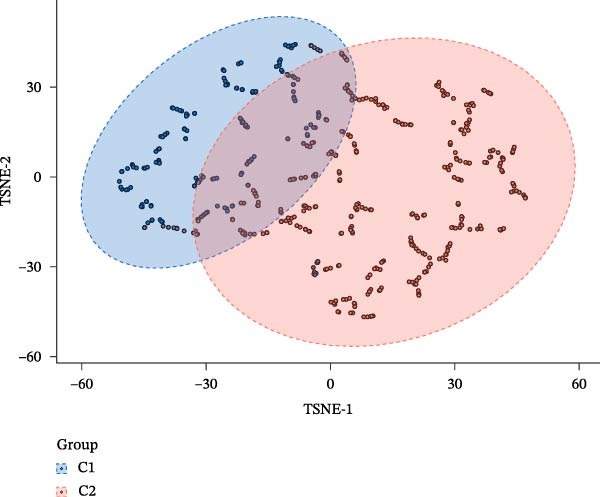
(B)

**Figure:**
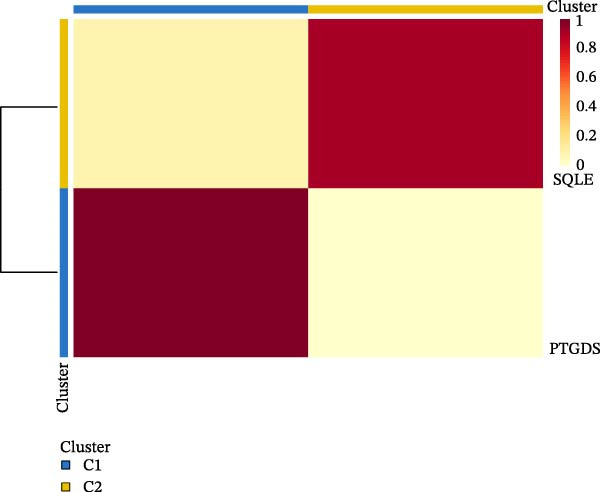
(C)

**Figure:**
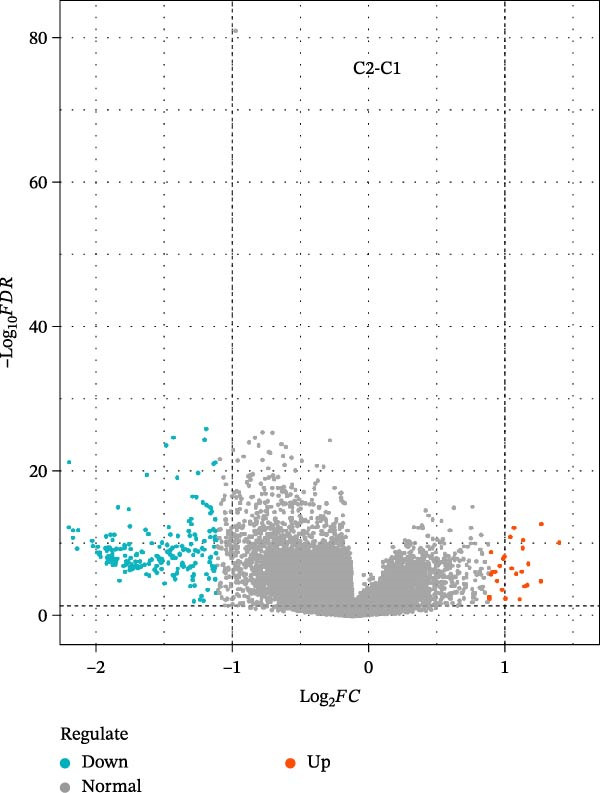
(D)

**Figure:**
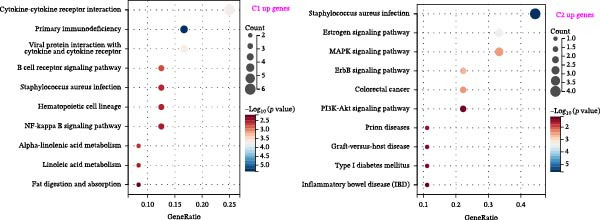
(E)

**Figure:**
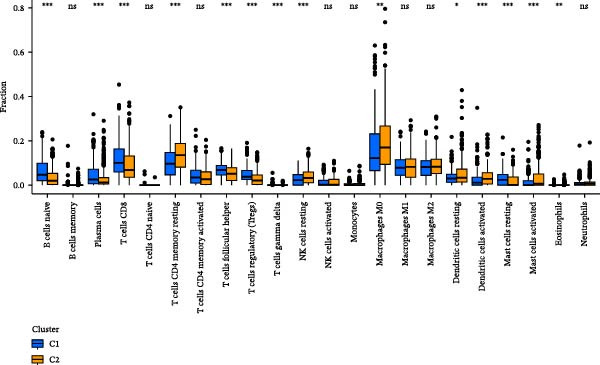
(F)

### 3.4. PTGDS Promoted Apoptosis and Inhibited the Malignant Phenotype of HNSCC

Given the pivotal role of PTGDS in regulating the immune microenvironment, we evaluated its cellular effects in vitro using Cal 27 and SCC 9 cell lines. First, we used qRT‐PCR to verify the transfection efficiency of PTGDS overexpression. PTGDS expression was significantly increased in the overexpression group (*p* < 0.05) (Figure 4A). Subsequently, CCK8 results showed that increased PTGDS expression significantly inhibited HNSCC cell proliferation (*p* < 0.05) (Figure 4B). Plate colony experiments showed that the colony‐forming ability of HNSCC cells overexpressing PTGDS was significantly reduced (*p* < 0.05) (Figure 4C). Transwell and scratch assays also showed that high PTGDS expression significantly inhibited the invasion and migration abilities of HNSCC cells (*p* < 0.05) (Figure 4D,E). Flow cytometry revealed that PTGDS overexpression significantly increased apoptosis in HNSCC cells (*p* < 0.05) (Figure 4F). These findings indicate that PTGDS functions as a tumor suppressor in HNSCC by inhibiting cell proliferation, invasion, and migration while promoting apoptosis (Figure 4G).

Figure 4Functional validation of PTGDS in HNSCC cell lines. (A) qRT‐PCR confirming PTGDS overexpression efficiency. (B) CCK8 assay showing reduced proliferation following PTGDS overexpression. (C) Colony formation assay demonstrating decreased clonogenic ability. Each colony‐forming unit was imaged from a field of view representing one 3 cm cell culture dish. Scale bars, 5 mm. (D) Transwell invasion and (E) wound‐healing assays reveal suppressed invasion and migration. Scale bars, 100 µm. (F) Flow cytometry analysis showing increased apoptosis in PTGDS‐overexpressing cells. (G) The quantification of colony formation, Transwell, wound healing, and flow cytometry analysis.

**Figure:**
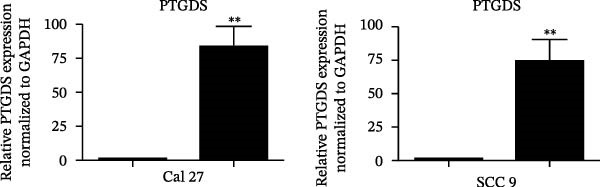
(A)

**Figure:**
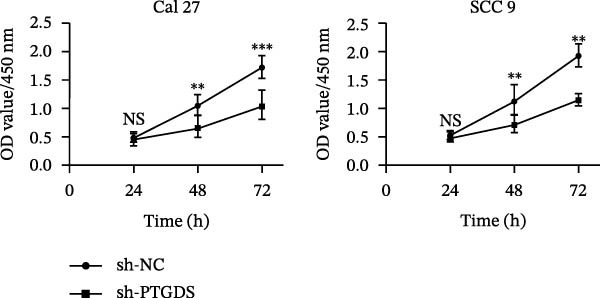
(B)

**Figure:**
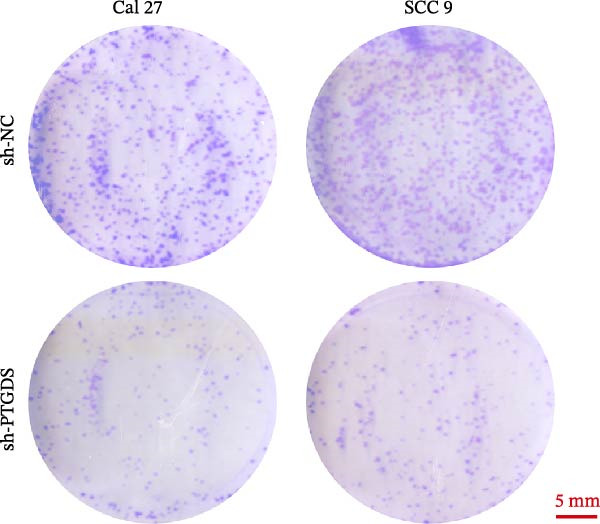
(C)

**Figure:**
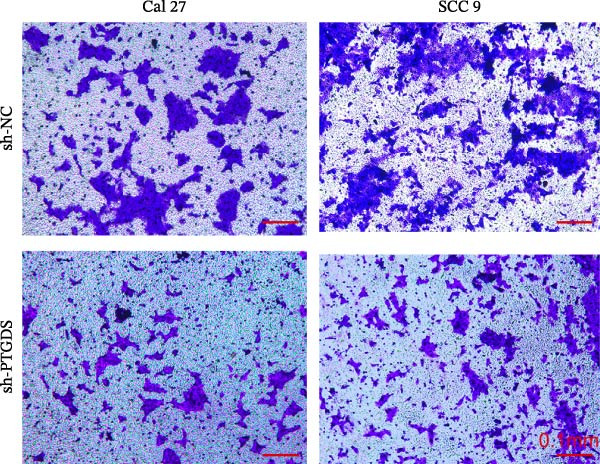
(D)

**Figure:**
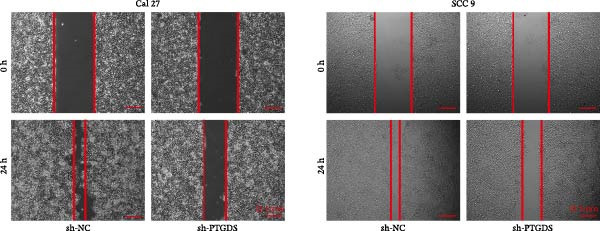
(E)

**Figure:**
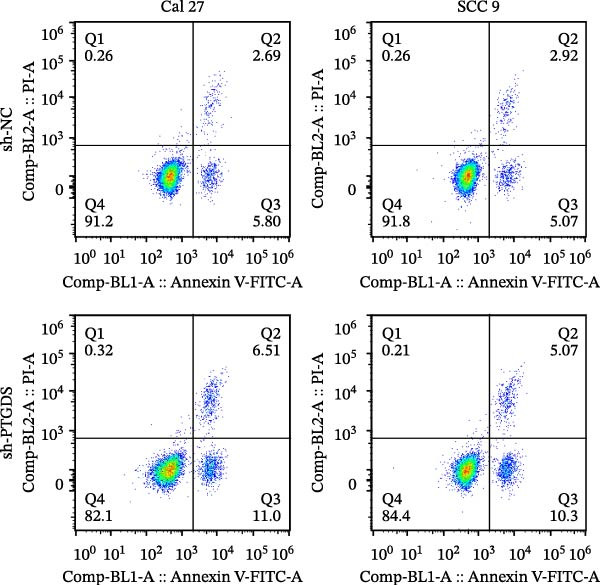
(F)

**Figure:**
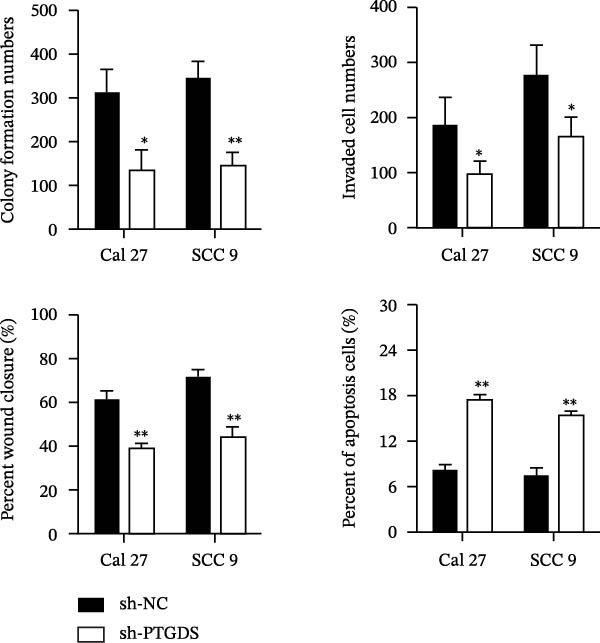
(G)

### 3.5. PTGDS Promoted CXCL13 Expression and Is Closely Related to Patients’ Clinicopathological Characteristics and Prognosis

To further investigate the relationship between PTGDS and CXCL13, we used Western blotting to find that overexpression of PTGDS significantly increased the expression level of CXCL13 (*p* < 0.05) (Figure 5A). Immunohistochemistry and prognostic analysis were used to further validate the roles of PTGDS and CXCL13. The results showed that PTGDS expression was generally weak and localized primarily to the intercellular matrix. Patients were divided into relatively high expression and relatively low expression groups based on the median expression value, with patients expressing relatively high PTGDS showing better prognosis (Figure 5B). CXCL13 was also stained and analyzed by immunohistochemistry; similarly, patients expressing high CXCL13 showed better prognosis (Figure 5C). Next, we assessed the association between PTGDS, CXCL13, and patient clinicopathological characteristics. The results showed that PTGDS expression correlated with primary tumor size, lymph node metastasis, TNM stage, and histological differentiation (Table 2). Similarly, the expression level of CXCL13 was closely related to the size of the primary tumor, lymph node metastasis, and TNM stage (Table 3). We used the chi‐square test to further verify the correlation between the expression of PTGDS and CXCL13 in the sections, and the results showed that the expression of the two in the sections was basically the same (*p* < 0.05) (Table 4).

Figure 5Clinical and pathological relevance of PTGDS and CXCL13 in HNSCC. (A) Western blot showing that PTGDS overexpression upregulates CXCL13 protein levels. (B) Immunohistochemical staining and survival analysis of PTGDS in HNSCC tissue sections. (C) Immunohistochemical staining and survival analysis of CXCL13 in HNSCC tissue sections. Scale bar, 100 µm. (D) Proposed working model of PTGDS.

**Figure:**
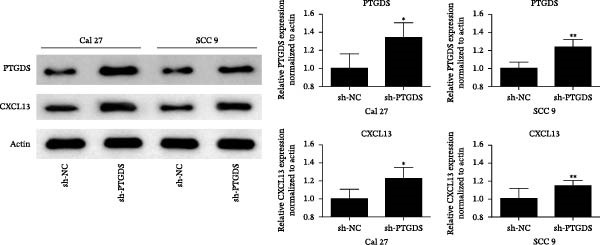
(A)

**Figure:**
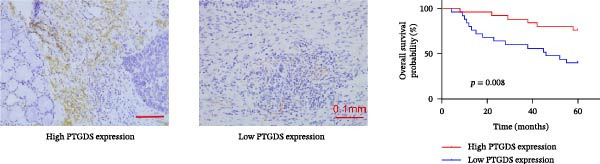
(B)

**Figure:**
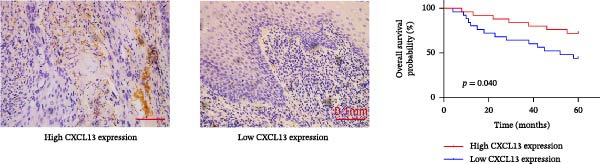
(C)

**Figure:**
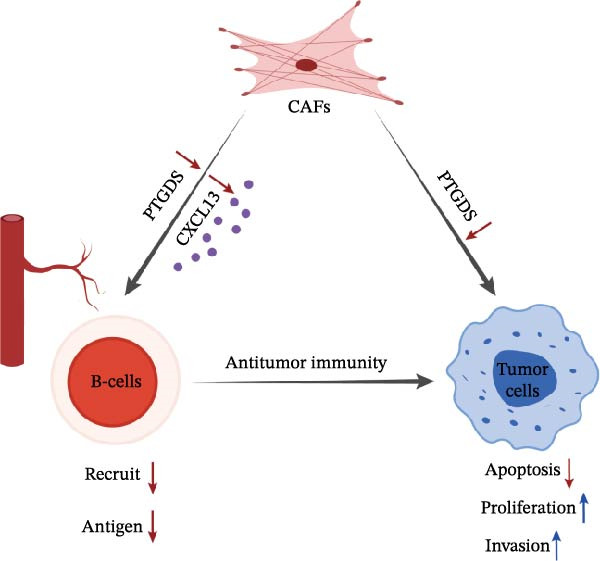
(D)

**Table 2:** Association between PTGDS expression and clinicopathological features in 50 primary HNSCC patients.

Feature	PTGDS		*χ* ^2^	*p*
	Low	High		
All	25	25	—	—
Age	—	—	0.80	0.37
>60	18	15	—	—
≤60	7	10	—	—
Gender	—	—	0.35	0.56
Male	17	15	—	—
Female	8	10	—	—
T category	—	—	10.78	**<0.01**
T1 + T2	11	22	—	—
T3 + T4	14	3	—	—
N category	—	—	4.37	**0.04**
N0	13	20	—	—
N1	12	5	—	—
TNM stage	—	—	8.68	**<0.01**
Stage I + stage II	11	21	—	—
Stage III + stage IV	14	4	—	—
Histology grade	—	—	4.16	**0.04**
Grade 1	12	19	—	—
Grade 2 + grade3	13	6	—	—

*Note:* N, lymph node; T, primary tumor growth. Bold values signify *p* < 0.05.

Abbreviations: *p*, *p*‐value; TNM, tumor‐node‐metastases.

**Table 3:** Association between CXCL13 expression and clinicopathological characteristics in 50 primary HNSCC patients.

Feature	CXCL13		*χ* ^2^	*p*
	Low	High		
All	25	25	—	—
Age	—	—	0.09	0.77
>60	16	17	—	—
≤60	9	8	—	—
Gender	—	—	1.39	0.24
Male	18	14	—	—
Female	7	11	—	—
T category	—	—	4.37	**0.04**
T1 + T2	13	20	—	—
T3 + T4	12	5	—	—
N category	—	—	7.22	**<0.01**
N0	14	19	—	—
N1	11	6	—	—
TNM stage	—	—	5.56	**0.02**
Stage I + stage II	12	20	—	—
Stage III + stage IV	13	5	—	—
Histology grade	—	—	2.12	0.15
Grade 1	13	18	—	—
Grade 2 + grade3	12	7	—	—

*Note:* N, lymph node; T, primary tumor growth. Bold values signify *p* < 0.05.

Abbreviations: *p*, *p*‐value; TNM, tumor‐node‐metastases.

**Table 4:** Correlation analysis between PTGDS and CXCL13 expression levels in HNSCC tissue samples.

Proteins		CXCL13		*χ* ^2^	*p*
		Low	High		
PTGDS	Low	46	4	70.56	<0.01
	High	4	46		

## 4. Discussion

HNSCC is the sixth most common malignant tumor worldwide. Due to its rapid cell growth, tendency to metastasize to lymph nodes, and high recurrence rate after surgery, it has gradually become a major public health concern globally [1]. The clinical stage of the tumor and the presence of lymph node metastasis are core factors determining patient prognosis. With the emergence of the TME, researchers have recognized that exploring the function and mechanism of proteins should be approached from the perspective of the TME. CAFs are central components of the TME, orchestrating processes such as matrix remodeling, apoptosis resistance, and immune evasion, thereby contributing to HNSCC progression, therapy resistance, and recurrence [27, 28]. CAFs originate from diverse cell types and display heterogeneous phenotypes and functions [29]. Similar to our previous research, using single‐cell sequencing data analysis, we first divided the CAF cell population into seven subpopulations [20]. Subsequently, we used pseudotemporal trajectory analysis to preliminarily clarify the potential differentiation directions of the seven cell subpopulations. This analysis also identified specific gene expression changes occurring during CAF differentiation.

PCD is a crucial pathway for the human body to eliminate abnormal cells and maintain tissue homeostasis. Tumors typically exhibit significant PCD disorder, leading to malignant biological behaviors such as excessive cell proliferation and metastasis [30]. Furthermore, the efficacy of radiotherapy and chemotherapy as comprehensive treatments for intermediate‐to‐advanced HNSCC is closely related to the PCD of tumor cells [31]. Studies have confirmed that the core mechanism of radiotherapy and chemotherapy resistance is the high expression of B cell CLL/lymphoma 2 (Bcl‐2) in tumor cells, which prevents the release of active cytochrome c from mitochondria, thereby significantly inhibiting apoptosis [32]. Using a list of PCD‐related genes compiled from the literature, we focused on exploring key proteins in the regulation of PCD by CAFs in HNSCC. Thirty‐two genes closely related to prognosis and exhibiting significant differential expression with CAF differentiation were named CAF‐related PCD regulatory proteins. Random forest is an ensemble learning algorithm based on decision trees, which improves accuracy and stability by combining the prediction results of multiple trees. We employed the random forest algorithm to evaluate the importance of these 32 key genes in the prognosis of HNSCC and confirmed that PTGDS, CDKN2A, and SQLE are the three most important genes determining the prognosis of HNSCC. TCGA cohort data and Kaplan–Meier assays were used to validate the prognostic value of the three genes. PTGDS was significantly underexpressed in HNSCC and showed a protective effect. SQLE, on the other hand, was significantly overexpressed in HNSCC and suggested a worse prognosis.

In recent years, immunotherapy has experienced rapid development and shown promising applications in various solid tumors [33]. For example, immune checkpoint inhibitors (PCD ligand 1, etc.) have demonstrated good efficacy in metastatic HNSCC and lung cancer [34]. However, immunotherapy exhibits significant individual variability, and some HNSCC patients fail to achieve adequate and durable efficacy. The response to immunotherapy often depends on the baseline level of immune cell infiltration in the TME [35]. Patients with high levels of immune cell infiltration (“hot tumors”) respond better to immunotherapy than those with low levels of immune cell infiltration (“cold tumors”) [36]. Therefore, we further evaluated the effects of PTGDS and SQLE on the abundance of immune cell infiltration in HNSCC. Our analyses revealed that elevated PTGDS expression correlated positively with increased infiltration of activated CD4^+^ and CD8^+^ T cells, supporting its potential role in enhancing antitumor immunity. Conversely, high SQLE expression was associated with an immunosuppressive TME. Simultaneously, we found that the expression levels, spatial distribution levels, and functions of PTGDS and SQLE appeared to show a significant negative correlation. To delineate the functional interplay between PTGDS and SQLE, TCGA patients were stratified into two expression‐defined subgroups for comparative analysis. The results showed that patients with high PTGDS expression were primarily enriched in immune cell‐related pathways, especially B cell–related signaling pathways.

Previous studies have relied on assessing T cell infiltration, particularly the well‐established crucial role of effector T cells in antitumor responses, while research on B lymphocytes has been less extensive. Recent studies have demonstrated that the presence and function of B cells can serve as important prognostic factors for tumors [37]. Active B cells (plasma cells) infiltrate tumor nests and produce large amounts of complement and cytokines. These components mediate antibody‐dependent cytotoxicity and phagocytosis, enhance antigen presentation, and strengthen antitumor immune responses [37, 38]. In this study, patients with elevated PTGDS expression demonstrated greater enrichment of naive and plasma B cells, suggesting a link between PTGDS activity and B cell–mediated immune regulation. Patients with high PTGDS expression also highly expressed genes such as CXCL13 and CD79A. Studies have confirmed that PTGDS promotes plasma cell infiltration into tertiary lymphoid structures in renal cell carcinoma via the CXCL12‐chemokine (C‐X‐C motif) receptor 4 (CXCR4) axis, thereby enhancing antitumor immunity [39]. Accumulating evidence indicates that B lymphocytes actively coordinate immune responses within the TME [40]. CXCL13 is a well‐known selective B cell chemokine, closely related to the infiltration level and function of B cells in tumor nests. We have reason to believe that CXCL13 may play an immunomodulatory role as a downstream gene of PTGDS.

Studies have shown that PTGDS expression gradually decreases during the progression of oral precancerous lesions. However, increased level of PGD2 can inhibit oral cancer cell proliferation by inducing G2 phase cell cycle arrest [16]. This may result from aberrant activation of the transforming growth factor‐β pathway, which reduces PTGDS expression and disrupts COX‐2 feedback regulation, leading to sustained COX‐2 activity and inflammation that promote oral cancer progression [16, 41]. However, the direct effects of PTGDS on oral cancer have not yet been reported. We subsequently supplemented our studies with in vitro cytology and immunohistochemistry of HNSCC sections to further clarify the biological function of PTGDS. The results showed that PTGDS expression significantly inhibited HNSCC cell proliferation, colony formation, invasion, and migration and promoted tumor cell apoptosis, which fully confirms that PTGDS can exert an antitumor effect. Through Western blotting experiments, we further found that high expression of PTGDS significantly increased the protein expression level of CXCL13. Immunohistochemical analysis of paraffin‐embedded tissue sections from HNSCC patients revealed that PTGDS expression levels were generally weak in HNSCC sections, concentrated in nonepithelial regions. Patients with relatively high PTGDS expression had better prognoses. PTGDS expression levels were closely related to primary tumor size, lymph node metastasis, TNM stage, and tumor differentiation degree.

## 5. Conclusions

Integrating bioinformatics, machine learning, phenotypic assays, and immunohistochemical analyses, our findings indicate that PTGDS may serve as a key CAF‐associated protein that enables tumor cells to evade PCD. This study preliminarily reveals several mechanisms by which PTGDS inhibits HNSCC: (1) PTGDS expression is significantly reduced in HNSCC development, which may significantly inhibit PCD in tumor cells; (2) low PTGDS expression promotes tumor cell clonogenicity, proliferation, migration, and invasion while also indicating a worse prognosis; (3) low PTGDS expression inhibits CXCL13 expression, resulting in reduced infiltration levels of immune cells (especially B lymphocytes) in HNSCC. These findings suggest that PTGDS may serve as a potential biomarker for early diagnosis and prognosis, as well as a therapeutic target for improving HNSCC outcomes (Figure 5D). However, this study has limitations, as it primarily relies on bioinformatic and machine learning analyses with limited experimental validation. The specific pathway mechanism by which PTGDS regulates CXCL13 remains unclear. It is crucial to further study PTGDS overexpression drugs and overcome the difficulties of drug penetration in vivo, clarify their more precise downstream targets and regulatory mechanisms, and verify the role of PTGDS overexpression in vivo.

## Author Contributions

Conceptualization: Dan Tao and Zhenxing Zhang. Formal analysis: Yuan Zhong and Haoran Zhu. Funding acquisition and project administration: Zhenxing Zhang. Supervision: Haoran Zhu. Writing – original draft: Dan Tao. Writing – review and editing: Dan Tao and Zhenxing Zhang.

## Funding

This study was supported by the Taizhou Social Development Science and Technology Project (Grant 23ywa20) and the Zhejiang Provincial Medical and Health Science and Technology Plan Project (Grant 2025KY1846).

## Disclosure

After using Grammarly, the authors reviewed and edited the content as needed and take full responsibility for the content of the publication. All authors have approved the manuscript and agree with its submission to Mediators of Inflammation.

## Ethics Statement

All procedures involving human participants in this study comply with the 1964 Helsinki Declaration and its subsequent amendments or similar ethical standards. This study has also been approved by the ethical standards of our institutional research committee (2020KT01).

## Consent

All patients involved in this study had given their informed consent before study. This work is original research and has not been submitted elsewhere for publication, in whole or in part.

## Conflicts of Interest

The authors declare no conflicts of interest.

## Supporting Information

Additional supporting information can be found online in the Supporting Information section.

## Supporting information


**Supporting Information 1** Figure S1. Molecular markers used for cell‐type clustering. (A) Marker genes defining major HNSCC cell clusters. (B) Marker genes defining CAF subclusters.

**Supporting Information 2** Figure S2. ssGSEA estimation of immune cell infiltration in HNSCC. Immune cell enrichment was evaluated based on median expression levels of (A) PTGDS, (B) CDKN2A, and (C) SQLE.

## Data Availability

The data that support the findings of this study are available from the corresponding author upon reasonable request. R software was used in this study. The relevant R package was explained in Section 2.

## References

[1] Johnson D. E. , Burtness B. , and Leemans C. R. , et al.Head and Neck Squamous Cell Carcinoma, Nature Reviews Disease Primers. (2020) 6, no. 1, 10.1038/s41572-020-00224-3, 92. PMC7944998 33243986

[2] Rasheduzzaman M. , Kulasinghe A. , and Dolcetti R. , et al.Protein Glycosylation in Head and Neck Cancers: From Diagnosis to Treatment, Biochimica et Biophysica Acta (BBA) - Reviews on Cancer. (2020) 1874, no. 2, 10.1016/j.bbcan.2020.188422, 188422. 32853734

[3] de Visser K. E. and Joyce J. A. , The Evolving Tumor Microenvironment: From Cancer Initiation to Metastatic Outgrowth, Cancer Cell. (2023) 41, no. 3, 374–403, 10.1016/j.ccell.2023.02.016. 36917948

[4] Ruffin A. T. , Li H. , Vujanovic L. , Zandberg D. P. , Ferris R. L. , and Bruno T. C. , Improving Head and Neck Cancer Therapies by Immunomodulation of the Tumour Microenvironment, Nature Reviews Cancer. (2023) 23, no. 3, 173–188, 10.1038/s41568-022-00531-9. 36456755 PMC9992112

[5] Guo Z. , Li K. , and Ren X. , et al.The Role of the Tumor Microenvironment in HNSCC Resistance and Targeted Therapy, Frontiers in Immunology. (2025) 16, no. 1, 10.3389/fimmu.2025.1554835. PMC11996806 40236700

[6] Kalluri R. , The Biology and Function of Fibroblasts in Cancer, Nature Reviews Cancer. (2016) 16, no. 9, 582–598, 10.1038/nrc.2016.73, 2-s2.0-84984694266. 27550820

[7] Li X. , González-Maroto C. , and Tavassoli M. , et al.Crosstalk Between CAFs and Tumour Cells in Head and Neck Cancer, Cell Death Discovery. (2024) 10, no. 1, 10.1038/s41420-024-02053-9, 303. 38926351 PMC11208506

[8] Desbois M. and Wang Y. , Cancer-Associated Fibroblasts: Key Players in Shaping the Tumor Immune Microenvironment, Immunological Reviews. (2021) 302, no. 1, 241–258, 10.1111/imr.12982. 34075584

[9] Newton K. , Strasser A. , and Kayagaki N. , et al.Cell Death, Cell. (2024) 187, no. 2, 235–256, 10.1016/j.cell.2023.11.044. 38242081

[10] Peng F. , Liao M. , and Qin R. , et al.Regulated Cell Death (RCD) in Cancer: Key Pathways and Targeted Therapies, Signal Transduction and Targeted Therapy. (2022) 7, no. 1, 10.1038/s41392-022-01110-y, 286. 35963853 PMC9376115

[11] Suresh V. , Dash P. , and Suklabaidya S. , et al.MIF Confers Survival Advantage to Pancreatic CAFs by Suppressing Interferon Pathway-Induced p53-Dependent Apoptosis, The FASEB Journal. (2022) 36, no. 8, 10.1096/fj.202101953R. 35839070

[12] Heichler C. , Scheibe K. , and Schmied A. , et al.STAT3 Activation Through IL-6/IL-11 in Cancer-Associated Fibroblasts Promotes Colorectal Tumour Development and Correlates With Poor Prognosis, Gut. (2020) 69, no. 7, 1269–1282, 10.1136/gutjnl-2019-319200. 31685519

[13] Dewey W. C. , Ling C. C. , and Meyn R. E. , Radiation-Induced Apoptosis: Relevance to Radiotherapy, International Journal of Radiation Oncology*Biology*Physics. (1995) 33, no. 4, 781–796, 10.1016/0360-3016(95)00214-8, 2-s2.0-0028846648. 7591884

[14] Hu S. , Ren S. , and Cai Y. , et al.Glycoprotein PTGDS Promotes Tumorigenesis of Diffuse Large B-Cell Lymphoma by MYH9-Mediated Regulation of Wnt-β-Catenin-STAT3 Signaling, Cell Death and Differentiation. (2022) 29, no. 3, 642–656, 10.1038/s41418-021-00880-2. 34743203 PMC8901925

[15] Li J. , Qu Z. , and Zhu D. , et al.Pan Cancer Research Reveals the Role of PTGDS in Tumor Suppression and Immune Regulation, npj Precision Oncology. (2025) 9, no. 1, 10.1038/s41698-025-01097-z, 319. 41028158 PMC12484698

[16] Banerjee AG. , Bhattacharyya I. , and Vishwanatha J. K. , Identification of Genes and Molecular Pathways Involved in the Progression of Premalignant Oral Epithelia, Molecular Cancer Therapeutics. (2005) 4, no. 6, 865–875, 10.1158/1535-7163.MCT-05-0033, 2-s2.0-21344453317. 15956244

[17] Liu Q. , Chen H. , and Tang D. , et al.Biological and Prognostic Insights Into the Prostaglandin D2 Signaling Axis in Lung Adenocarcinoma, Frontiers in Pharmacology. (2025) 16, no. 1, 10.3389/fphar.2025.1562261. PMC12138261 40474982

[18] Zhang Q. , Wang F. , and Huang Y. , et al.PGD2/PTGDR2 Signal Affects the Viability, Invasion, Apoptosis, and Stemness of Gastric Cancer Stem Cells and Prevents the Progression of Gastric Cancer, Combinatorial Chemistry & High Throughput Screening. (2024) 27, no. 6, 933–946, 10.2174/1386207326666230731103112. 37526190

[19] Hao Y. , Hao S. , and Andersen-Nissen E. , et al.Integrated Analysis of Multimodal Single-Cell Data, Cell. (2021) 184, no. 13, 3573–3587, 10.1016/j.cell.2021.04.048. 34062119 PMC8238499

[20] Mou T. , Zhu H. , and Jiang Y. , et al.Heterogeneity of Cancer-Associated Fibroblasts in Head and Neck Squamous Cell Carcinoma, Translational Oncology. (2023) 35, no. 10, 10.1016/j.tranon.2023.101717, 101717. 37320872 PMC10277597

[21] Zou Y. , Xie J. , and Zheng S. , et al.Leveraging Diverse Cell-Death Patterns to Predict the Prognosis and Drug Sensitivity of Triple-Negative Breast Cancer Patients After Surgery, International Journal of Surgery. (2022) 107, no. 1, 10.1016/j.ijsu.2022.106936, 106936. 36341760

[22] Qiu X. , Hill A. , and Packer J. , et al.Single-Cell mRNA Quantification and Differential Analysis With Census, Nature Methods. (2017) 14, no. 3, 309–315, 10.1038/nmeth.4150, 2-s2.0-85010878111. 28114287 PMC5330805

[23] Liaw A. and Wiener M. , Classification and Regression by Random Forest, R News. (2002) 2, no. 3, 18–22.

[24] Bergenstråhle J. , Larsson L. , and Lundeberg J. , Seamless Integration of Image and Molecular Analysis for Spatial Transcriptomics Workflows, BMC Genomics. (2020) 21, no. 1, 10.1186/s12864-020-06832-3, 482. 32664861 PMC7386244

[25] Hänzelmann S. , Castelo R. , and Guinney J. , GSVA: Gene Set Variation Analysis for Microarray and RNA-Seq Data, BMC Bioinformatics. (2013) 14, no. 1, 10.1186/1471-2105-14-7, 2-s2.0-84872202078. PMC3618321 23323831

[26] Jia Q. , Wu W. , and Wang Y. , et al.Local Mutational Diversity Drives Intratumoral Immune Heterogeneity in Non-Small Cell Lung Cancer, Nature Communications. (2018) 9, no. 1, 10.1038/s41467-018-07767-w, 2-s2.0-85058756046, 5361. PMC6299138 30560866

[27] Jia H. , Chen X. , and Zhang L. , et al.Cancer Associated Fibroblasts in Cancer Development and Therapy, Journal of Hematology & Oncology. (2025) 18, no. 1, 10.1186/s13045-025-01688-0, 36. 40156055 PMC11954198

[28] Zhang H. , Yue X. , and Chen Z. , et al.Define Cancer-Associated Fibroblasts (CAFs) in the Tumor Microenvironment: New Opportunities in Cancer Immunotherapy and Advances in Clinical Trials, Molecular Cancer. (2023) 22, no. 1, 10.1186/s12943-023-01860-5, 159. 37784082 PMC10544417

[29] Wu F. , Yang J. , and Liu J. , et al.Signaling Pathways in Cancer-Associated Fibroblasts and Targeted Therapy for Cancer, Signal Transduction and Targeted Therapy. (2021) 6, no. 1, 10.1038/s41392-021-00641-0, 218. 34108441 PMC8190181

[30] Dai X. , Wang D. , and Zhang J. , Programmed Cell Death, Redox Imbalance, and Cancer Therapeutics, Apoptosis. (2021) 26, no. 7, 385–414, 10.1007/s10495-021-01682-0. 34236569

[31] O’Leary B. , Skinner H. , and Schoenfeld J. D. , et al.Evasion of Apoptosis and Treatment Resistance in Squamous Cell Carcinoma of the Head and Neck, Cancer Treatment Reviews. (2024) 129, no. 10, 10.1016/j.ctrv.2024.102773, 102773. 38878677

[32] Ryan C. E. and Davids M. S. , BCL-2 Inhibitors, Present and Future, Cancer Journal. (2019) 25, no. 6, 401–409, 10.1097/PPO.0000000000000408. 31764121

[33] Riley R. S. , June C. H. , Langer R. , and Mitchell M. J. , Delivery Technologies for Cancer Immunotherapy, Nature Reviews Drug Discovery. (2019) 18, no. 3, 175–196, 10.1038/s41573-018-0006-z, 2-s2.0-85059772897. 30622344 PMC6410566

[34] Wu X. , Gu Z. , and Chen Y. , et al.Application of PD-1 Blockade in Cancer Immunotherapy, Computational and Structural Biotechnology Journal. (2019) 17, no. 6, 661–674, 10.1016/j.csbj.2019.03.006, 2-s2.0-85066755088. 31205619 PMC6558092

[35] Chen Y. , Li Z.-Y. , Zhou G.-Q. , and Sun Y. , An Immune-Related Gene Prognostic Index for Head and Neck Squamous Cell Carcinoma, Clinical Cancer Research. (2021) 27, no. 1, 330–341, 10.1158/1078-0432.CCR-20-2166. 33097495

[36] Wu B. , Zhang B. , and Li B. , et al.Cold and Hot Tumors: From Molecular Mechanisms to Targeted Therapy, Signal Transduction and Targeted Therapy. (2024) 9, no. 1, 10.1038/s41392-024-01979-x, 274. 39420203 PMC11491057

[37] Fitzsimons E. , Qian D. , and Enica A. , et al.A Pan-Cancer Single-Cell RNA-Seq Atlas of Intratumoral B Cells, Cancer Cell. (2024) 42, no. 10, 1784–1797, 10.1016/j.ccell.2024.09.011. 39406187

[38] Lauss M. , Donia M. , and Svane IM. , et al.B Cells and Tertiary Lymphoid Structures: Friends or Foes in Cancer Immunotherapy?, Clinical Cancer Research. (2022) 28, no. 9, 1751–1758, 10.1158/1078-0432.CCR-21-1130. 34965949 PMC9306440

[39] Meylan M. , Petitprez F. , and Becht E. , et al.Tertiary Lymphoid Structures Generate and Propagate Anti-Tumor Antibody-Producing Plasma Cells in Renal Cell Cancer, Immunity. (2022) 55, no. 3, 527–541, 10.1016/j.immuni.2022.02.001. 35231421

[40] Downs-Canner S. M. , Meier J. , Vincent B. G. , and Serody J. S. , B Cell Function in the Tumor Microenvironment, Annual Review of Immunology. (2022) 40, no. 1, 169–193, 10.1146/annurev-immunol-101220-015603. 35044794

[41] Hsu S. , Borke J. L. , and Lewis J. B. , et al.Transforming Growth Factor Beta 1 Dysregulation in a Human Oral Carcinoma Tumour Progression Model, Cell Proliferation. (2002) 35, no. 3, 183–192, 10.1046/j.1365-2184.2002.00237.x, 2-s2.0-18344386381. 12027954 PMC6496909

