# Supplementary figures and images for: Low PTGDS Expression Facilitates HNSCC by Suppressing Programmed Cell Death and Reducing B Cell–Mediated Immune Responses
Source: Mediators Inflamm. 2026 Mar 22;2026:4521847. doi: 10.1155/mi/4521847 (PMC13140297; doi:10.1155/mi/4521847)

(a)

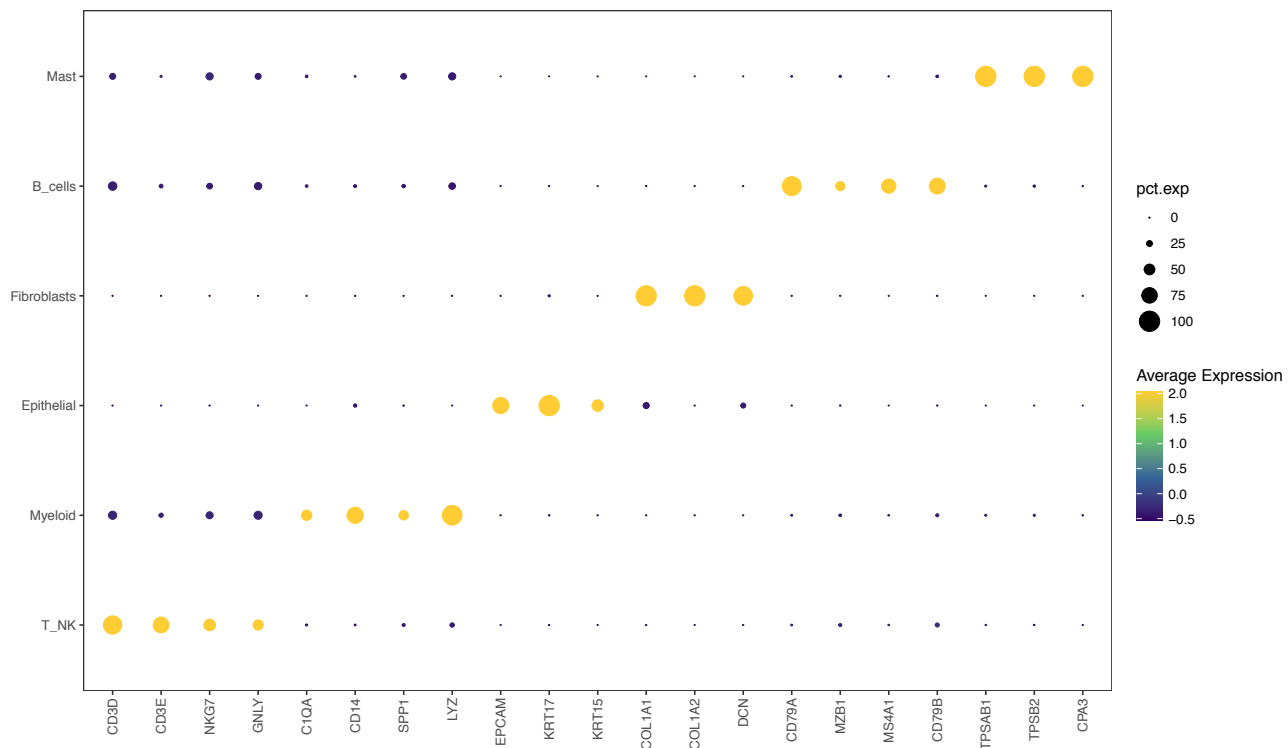

(b)

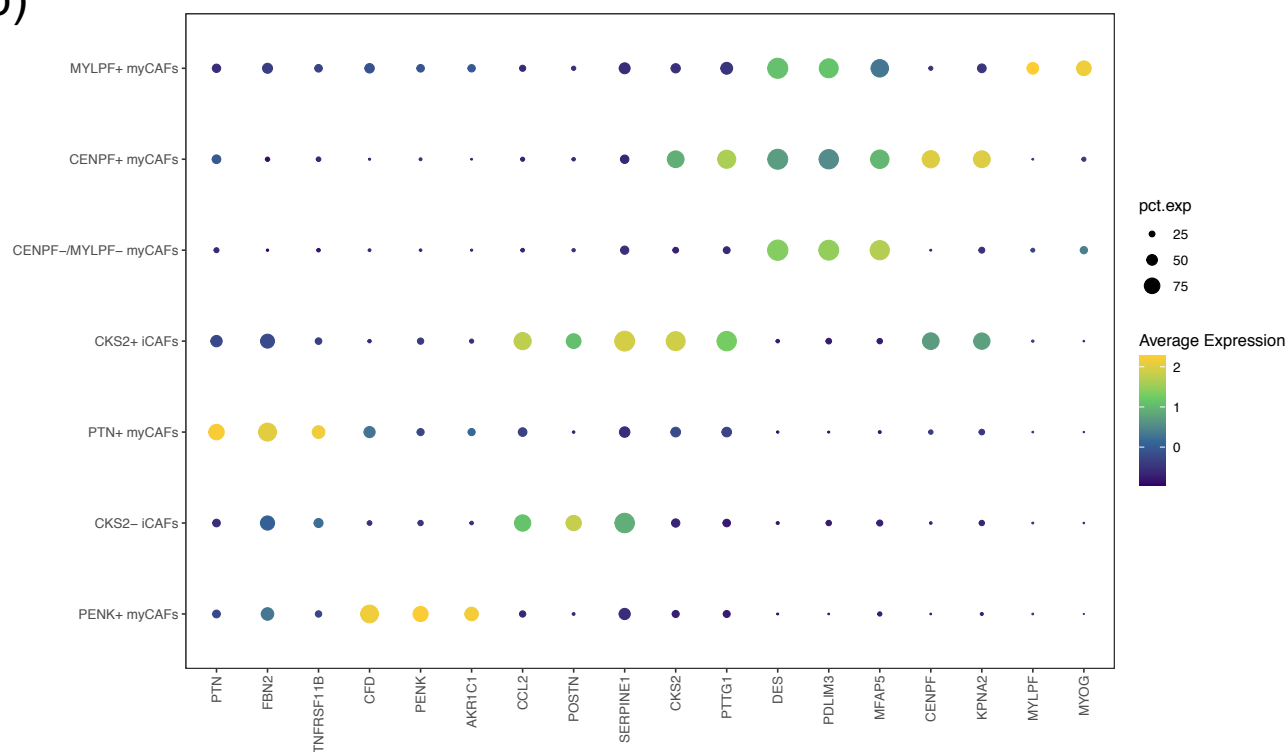

Supplement: Supplementary file 1 — Supporting Information 1 Figure S1. Molecular markers used for cell‐type clustering. (A) Marker genes defining major HNSCC cell clusters. (B) Marker genes defining CAF subclusters. [file MI-2026-4521847-s001.pdf]

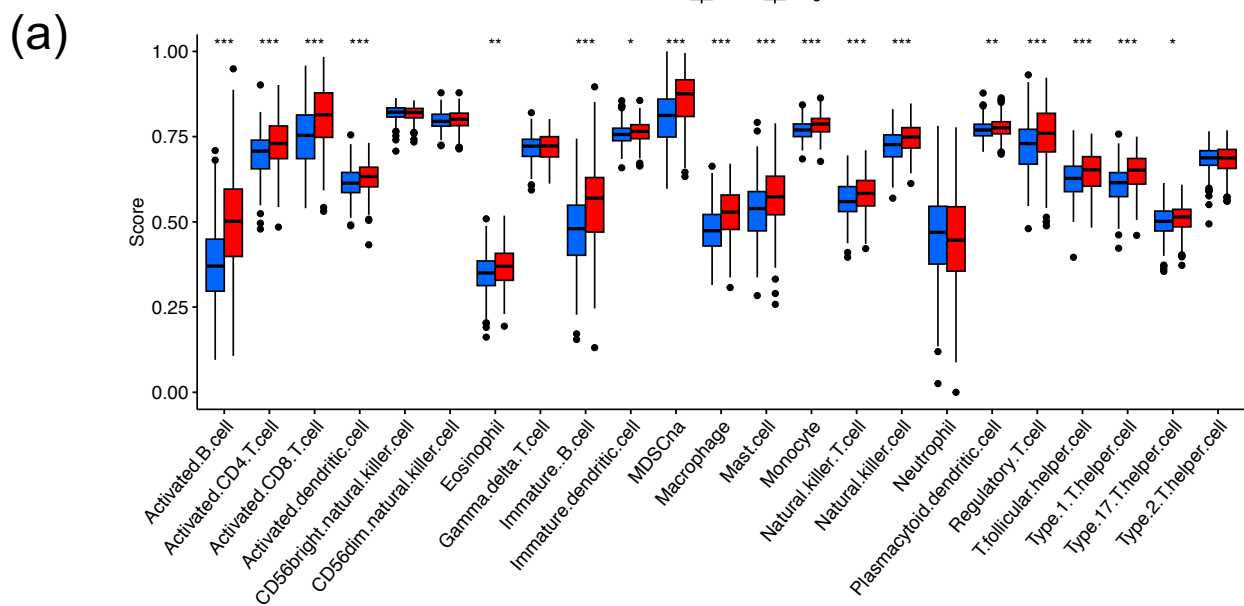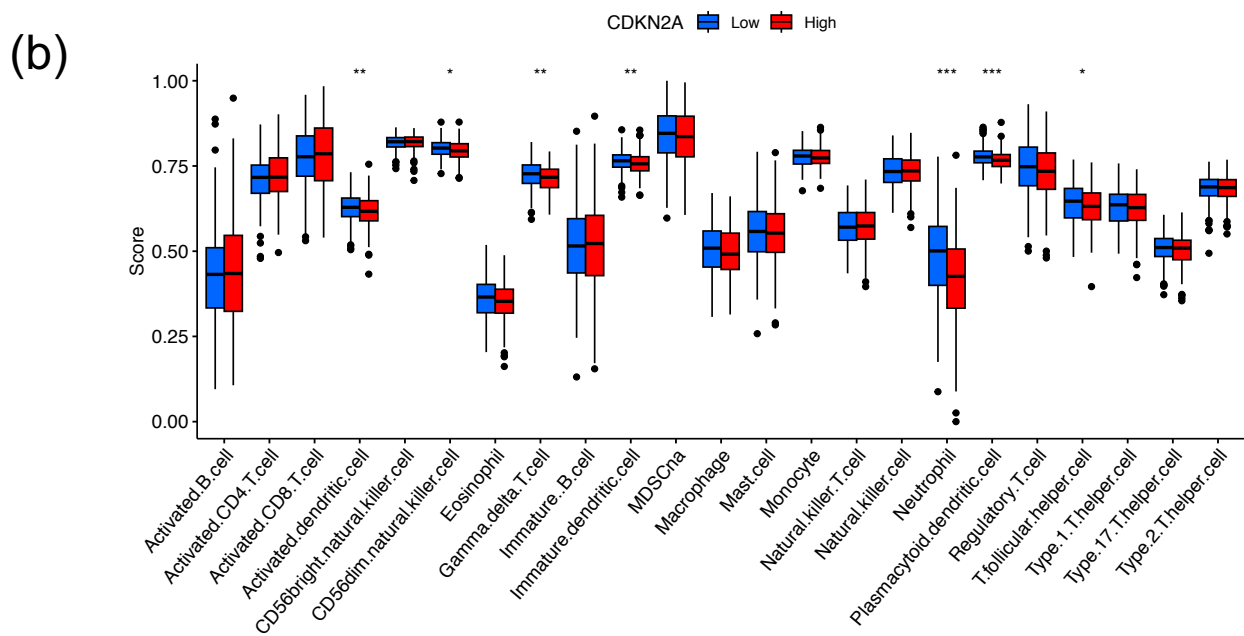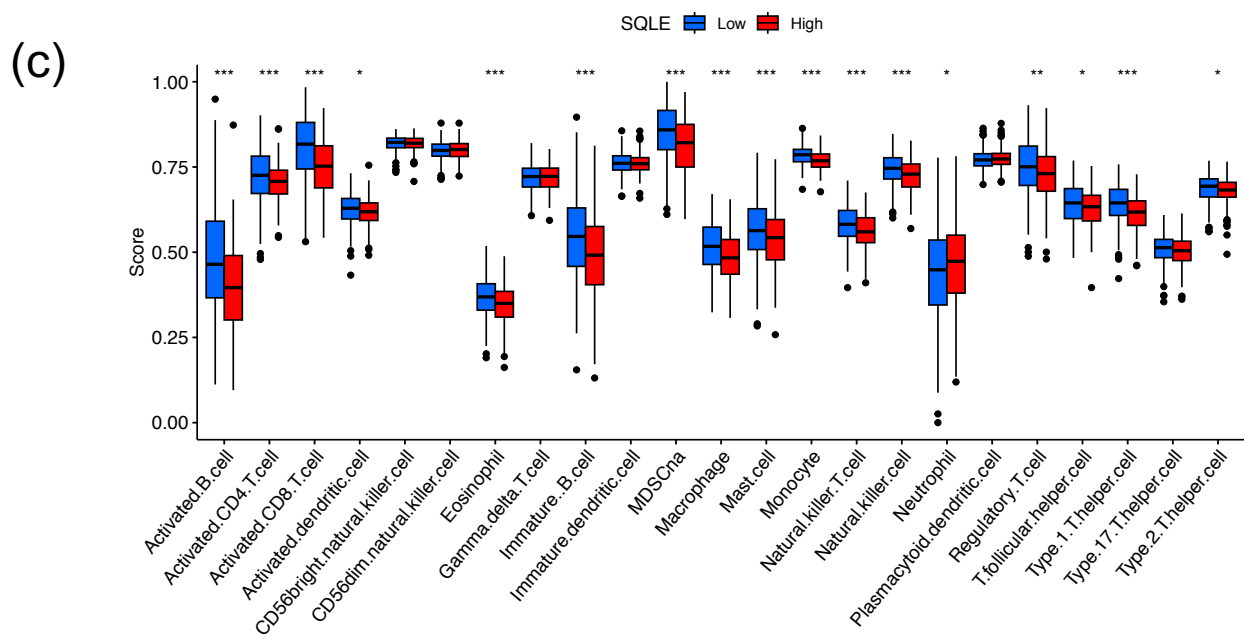

Supplement: Supplementary file 2 — Supporting Information 2 Figure S2. ssGSEA estimation of immune cell infiltration in HNSCC. Immune cell enrichment was evaluated based on median expression levels of (A) PTGDS, (B) CDKN2A, and (C) SQLE. [file MI-2026-4521847-s002.pdf]
